# Proteomics for blood biomarker exploration of severe mental illness: pitfalls of the past and potential for the future

**DOI:** 10.1038/s41398-018-0219-2

**Published:** 2018-08-16

**Authors:** Ashley L. Comes, Sergi Papiol, Thorsten Mueller, Philipp E. Geyer, Matthias Mann, Thomas G. Schulze

**Affiliations:** 1 Institute of Psychiatric Phenomics and Genomics (IPPG), University Hospital Munich, LMU, 80336 Munich, Germany; 2 International Max Planck Research School for Translational Psychiatry (IMPRS-TP), 80804 Munich, Germany; 3 Department of Psychiatry and Psychotherapy, University Hospital, Ludwig Maximilian University, 80336 Munich, Germany; 4 Department of Proteomics and Signal Transduction, Max Planck Institute of Biochemistry, Martinsried, Germany; 5 NNF Center for Protein Research, Faculty of Health Sciences, University of Copenhagen, Copenhagen, Denmark

## Abstract

Recent improvements in high-throughput proteomic approaches are likely to constitute an essential advance in biomarker discovery, holding promise for improved personalized care and drug development. These methodologies have been applied to study multivariate protein patterns and provide valuable data of peripheral tissues. To highlight findings of the last decade for three of the most common psychiatric disorders, namely schizophrenia (SZ), bipolar disorder (BD), and major depressive disorder (MDD), we queried PubMed. Here we delve into the findings from thirty studies, which used proteomics and multiplex immunoassay approaches for peripheral blood biomarker exploration. In an explorative approach, we ran enrichment analyses in peripheral blood according to these results and ascertained the overlap between proteomic findings and genetic *loci* identified in genome-wide association studies (GWAS). The studies we appraised demonstrate that proteomics for psychiatric research has been heterogeneous in aims and methods and limited by insufficient sample sizes, poorly defined case definitions, methodological inhomogeneity, and confounding results constraining the conclusions that can be extracted from them. Here, we discuss possibilities for overcoming methodological challenges for the implementation of proteomic signatures in psychiatric diagnosis and offer an outlook for future investigations. To fulfill the promise of proteomics in mental disease diagnostics, future research will need large, well-defined cohorts in combination with state-of-the-art technologies.

## Introduction

Psychiatric disorders, such as schizophrenia (SZ), bipolar disorder (BD), and major depressive disorder (MDD), are severe mental illnesses associated with morbidity and life-long disability for sufferers^1–6^. Our understanding of their etiology and pathophysiology remains incomplete. Being that, each is defined by a spectrum of heterogeneous signs and symptoms, often overlapping across disorders, biological investigations have been hindered. The complex interplay between social, psychological, etiological, and environmental factors – combined with the difficulty in generating accurate animal models, has complicated molecular and mechanistic studies. As a result, reliable biomarkers related to the prognosis and diagnosis of these patients remain an unmet clinical need. One of the most remarkable consequences in the day-to-day clinical practice is that treatment selections is based on descriptive psychopathology, contributing to low therapeutic effectiveness^7^. The consequence is the significant proportion of disease burden and health costs worldwide associated with SZ, BD, and MDD^8^. Global direct and indirect economic costs of mental disorders were estimated at US$2.5 trillion, based on 2010 data, therefore accounting for more economic costs than chronic somatic diseases like cancer or diabetes. These estimates are expected to double by 2030^9^. Advances in technologies allowing high-throughput biological analyses have introduced new opportunities for a better understanding of these burdensome disorders. Such investigations hold the promise for the discovery of biomarkers that could be applied, especially by minimally invasive approaches, as predictors concerning diagnosis and outcomes. These predictors would enable earlier, more effective care for patients, a better allocation of the resources of the health system and clues to the underlying biological mechanisms of disease course.

## The promise of proteomics for biomarker identification

According to the Biomarkers Definitions Working Group, a biological marker (biomarker) is “a characteristic that is objectively measured and evaluated as an indicator of normal biological processes, pathogenic processes, or pharmacologic response to a therapeutic intervention.”^10^ Over the last years, there has been an interest in incorporating biomarkers into psychiatry. Accordingly, first attempts have focused on genomic analyses, which until now have offered little in the way of reliable biomarkers^11^, especially to “differentiate between similar phenotypes and disease states, to monitor therapeutic progress or to assess the prognosis of individual patients.”^12^

Hypothesis-driven efforts identified genetic trait and state biomarkers for MDD with little success in identifying reliable molecular risk factors^13,14^. For bipolar disorder, robust and replicable associations have been reported in GWAS-studies, many of which have shown evidence for an overlap of susceptibility between BD and SZ^15^. While an overlap in risk alleles is clear, this does not imply homogeneity^16^ and it should be recognized that an absence of genetic association signals does not mean that a gene’s protein product does not play an important role in disease pathogenesis^15^. With the identification of numerous varied loci, GWAS studies highlighted the importance of exploring pathways and circuits rather than single gene products and pushed for biological information like protein expression and biological pathways to be integrated with genetic data^15^. Further investigations focusing on the protein expression level will shed light on the functional role of these risk loci to determine how these associations map on to particular endophenotypes that could be useful for classifying the disorders^16^. Towards this cause, researchers have started looking at molecular levels closely tied to the phenotype of an individual. Proteins perform the vast majority of functions in every organism, making them potentially more useful to translational approaches than the genome or transcriptome.

The proteome is the entire set of proteins produced or modified by an organism and varies with time, biological requirements, stress, and other environmental factors^17^. An intriguing example is the butterfly who shares the same genome with the caterpillar but whose phenotypical differences are clearly due to differences in proteome patterns. Proteomics refers to a large-scale and global analysis of the proteins in a system, at a specific point in time under a determined condition^12^. It aims to “obtain a more global and integrated view of biology by studying all the proteins of a cell rather than each one individually*”*^12,17^. Thus, protein profiling may better reflect dynamic pathophysiological processes. Notable is the fact that proteomics is uniquely capable of representing both expression levels of proteins and the isoform (or ‘proteoforms’), as well as their post-translational modifications^18^.

In the last years, the development of high-throughput technologies of proteomic analysis has introduced a new era of biomarker discovery. For complex, multifactorial disorders, ‘molecular fingerprinting’ via the identification and characterization of biomarker profiles has enabled greater diagnostic resolution between closely related disease phenotypes^19^. For psychiatric disorders, like SZ, BD, and MDD, such profiling allows for the generation of predictive models regardless of the disease causes, which generally remain largely unknown. Furthermore, it holds promise not only for predicting the onset of a disorder but also its course and outcome^20^.

## An overview of proteomic methodologies for biomarker discovery

Since the term proteomics was coined in the late 90 s, several methods have been employed to study proteins^21^. In general, antibody-based methods (immunoassays) or mass-spectrometry (MS) are used for protein detection. Enzyme-linked immunosorbent assays (ELISA) and Western blots have been applied for decades as validation tools to detect and quantify candidate proteins. In the last years, higher-throughput multiplex immunoassay panels have been developed to simultaneously identify and quantify hundreds of proteins. While immunoassay methods have matured over the last decades, these methods face inherent limitations with regard to multiplexing, specificity for protein isoforms and incompatibility with hypothesis-free investigations^22,23^. In this regard MS-based methods have become advantageous. With technological developments over the past years dramatically improving MS-based proteomics, these methods can now characterize human plasma proteomes with unprecedented accuracy^24^.

In mass spectrometry (MS)-based proteomics, there are multiple methods of protein separation, visualization, and analysis. In contrast to immunoassays, proteins are detected using mass spectrometry instruments. In the first decade of proteomics, gel-based two-dimensional electrophoresis (2DE), including fluorescent two-dimensional differential gel electrophoresis (2D-DIGE), were the main methods used for relative quantification of protein abundances between samples^25^. However, as such gel-based approaches are labor-intensive, limited by poor separation of certain protein groups (especially membrane proteins) and generally only identified a small number of proteins, these methods never fulfilled their aim of large-scale proteome characterization. Today, the most widespread workflow for discovery proteomics is termed shotgun proteomics^26^. It starts with a sample preparation step, in which a complex protein mixture is enzymatically digested into peptides. This step is followed by a combination of a liquid chromatography (LC) system that allows the separation of the peptides over time and the ionization by the electrospray ionization technology, for which the Nobel Prize was awarded in 2001 to John Fenn^27^. It allows the formation of charged molecules, followed by their analysis in the mass spectrometer (LC-MS). Peptides are fragmented in and the results of these MS/MS spectra run through sequence databases by ‘search engines’ to identify peptides using statistically defined criteria^28^. In contrast, in ‘targeted proteomics’ data on a relatively small number of peptides of interest (typically far less than 100) are acquired with high specificity and sensitivity^29^. The most common targeted MS method is termed ´multiple reaction monitoring´ (MRM). For a selected set of targeted peptides, a higher sensitivity and throughput may be achievable compared to shotgun proteomics. For both shotgun and targeted proteomics, including heavy isotope labeled peptides as reference standards for endogenous peptides of interest enables absolute protein quantification.

## A systematic approach for identifying the potential and pitfalls of proteomics studies in psychiatry

For clinical use, proteomics approaches using blood, plasma or serum would be a highly desired method for biomarker profiling of psychiatric disorders. Not only are these biological samples well established for use in diagnostic analyses in clinical practice, but they are readily available in biobanks across thousands of clinical studies^30,31^. For such reasons, here we have taken a systematic approach to obtain a comprehensive view of such minimally invasive proteomics studies in SZ, BD, and MDD, by reviewing studies from the last decade. Accordingly, we used these studies to acknowledge what proteomics investigations have been able to uncover so far and to guide a perspective on overcoming limitations of proteomics in psychiatric research. In the last few years, a number of exhaustive reviews have been published^11,12,22,25,32,33^. Here we narrow the focus to evaluate and connect those studies using peripheral blood of patients with three of the most common psychiatric disorders.

**Fig. 1:**
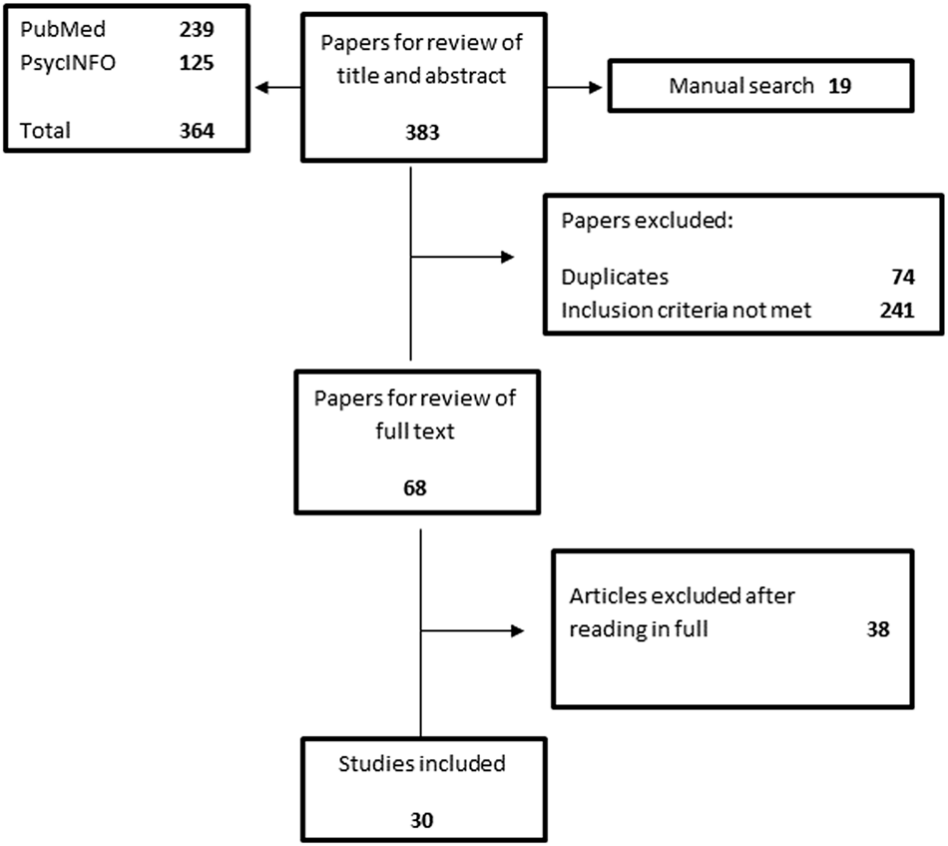
Flow diagram depicting the flow of information through the different phases of review

We queried PubMed with the search syntax “(proteomic OR proteome profiling) AND (schizophrenia OR schizoaffective disorder OR schizophreniform disorder OR bipolar disorder OR major depression).” Publications containing original data on proteomic biomarkers in the diagnosis, risk stratification or differentiation of individuals with a DSM-IV or ICD-10 diagnosis of SZ, schizoaffective disorder (SZA), BD-I/II, and/or MDD were considered of interest. This yielded initially 388 studies. All animal studies were excluded. The number of studies was further reduced by screening the literature to include only those studies that were (1) non-interventional, (2) used minimally invasive samples, here defined as blood, plasma and/or serum samples, and (3) which used standard MS-based proteomic methods or multi-target immunoassays i.e., excluding validation studies using single analyte ELISA or Western blot methods only (see Fig. 1). This left thirty papers for original data abstraction and review of findings (see Table 1).

**Table 1:** Summary of included studies. Summary of all included studies in terms of size, sample type, techniques used, number of altered proteins and major findings

Authors	Study subjects (*n*, clinical diagnosis)	Sample (enrichment/ depletion)	Technique	Number of proteins with altered abundance	Findings^a^
^47^	39, male MDD; 30, male controls	Blood serum (C8 protein-, C18 peptide)	MALDI	3 peptide signals offered greatest potential as biomarkers	Combined *m/z* 1017, *m/z* 1042, and *m/z* 1479 offered the most specificity and sensitivity identifying true (+) depression cases
^34^	26, SZ; 26, controls	Blood serum (C8 protein-, C18 peptide-, TiO_2_ phosphopeptide)	MALDI	16 proteins/phosphoproteins	Combined metabolome/proteome profiling: Ion at *m*/*z* 3177 reduced in cases (suspected to be Apolipoprotein A-I) considered prospective biomarker
^52^	24, BD; 21, controls	Blood serum and plasma	Multiplex immunoassay	6 proteins in serum 10 proteins in plasma	Expressed differences in proteome coverage/reliability of measurement when comparing serum and plasma analyses
^18^	687, MDD (current); 482, MDD (remitted); 420, controls	Blood serum	Multiplex immunoassay	28 different between cMDD and controls; Additional 6 different between rMDD and controls (4 overlapped)	Analytes prominently associated with cMDD related to diverse cell communication and signal transduction processes, immune response and protein metabolism
^57^	20, BD-II; 30, MDD; 30, controls	Plasma	2-DE coupled with MALDI-TOF MS/MS; ELISA for validation	25 with at least 2-fold differences in BD II versus MDD; 3 proteins significantly differentially expressed in all cases versus controls	The most significantly enriched biological processes based on the 25 differentially expressed proteins identified in BD-II relative to MDD were immune regulatory, including defense response, acute inflammatory response, innate immune response, response to wounding and inflammatory response
^35^	42, male first-onset SZ; 31, male controls	Blood serum	Human Metabolic MAP platform	9	Differentially expressed molecules involved in regulation of metabolic signaling pathway
^48^	80, remitted LLD (36 with mild cognitive impairment and 44 with normal cognitive function)	Plasma	Multiplex immunoassay	24	Proteins significantly associated with cognitive impairment in LLD related to inflammatory cascade, trophic factors, and nutrient sensing and insulin signaling cascades
^58^	229, SZ; 245, MDD; 254, controls	Plasma	Multiplex immunoassay	79	Proteins involved in synaptic transmission, growth factors, chemo-attractants, proteolytic system. Insulin was the marker with the highest statistically significant finding
^53^	52, Unipolar; 49, BD-II; 46, BD-I; 141, controls	Blood serum	Multiplex immunoassay	73 showed nominally significant differences; 6 different after Bonferroni correction	Differentially expressed proteins involved in a range of processes including brain maturation and cognition, acquisition of memory and behavioral activities
^59^	66, first-onset/acutely psychotic SZ; 10 euthymic BD; 78 controls	Blood serum	Fluorescence assays and immunoassay	5	Dysregulation of glucose metabolic pathways. Suggests insulin-related molecules and potentially other co-secreted insulin-secretory granule proteins may be potential biomarkers
^36^	236, first-/recent-onset SZ; 230, controls	Blood serum	Metabolic MAP multiplexed immunoassay/2D-DIGE	7	Dysregulation of HPA axis hormones and glucose metabolism, supporting hypothesis of metabolic unbalance
^54^	17, outpatient BD; 46, controls	Plasma	Human DiscoveryMAP multiplexed immunoassay	26	Differentially expressed proteins comprised mostly of growth factors, hormones, lipid transport and inflammatory proteins
^37^	17, first-episode/paranoid SZ; 17, controls	Blood serum	HumanMAP multiplexed immunoassay	9 in cohort 1; 22 in cohort 2 (data not shown)	Differentially expressed proteins have roles in endothelial cell function, inflammation, acute phase response and fibrinolysis pathways
^55^	16, euthymic BD-I outpatients (remitted); 16, euthymic BD-II outpatients (remitted); 32, controls Validation cohort: 7, BD-I; 7, BD-II; 14, controls	Blood serum (and PBMCs)	Multi-Analyte Profiling (Human Map)	60	Differentially expressed proteins involved predominantly in cell death/survival pathways
^39^	22, first-onset/paranoid SZ; 33, controls	Serum	Label-free nano LC-MS^E^, > 2 proteins per peptide	10 phosphopeptides corresponding to 8 distinct proteins	Altered proteins were inflammatory, serine-type endopeptidase inhibitors and structural proteins
^38^	20, first-onset SZ; 20, controls	Blood serum, depletion of 14 high abundant proteins)	IMAC/LC-MS/MS (MS^E^), application of decoy database	35	Differentially expressed proteins involved in several processes and pathways including acute phase response, complement system, coagulation system, LXR/RXR activation, and intrinsic prothrombin activation pathway
^49^	25, female MDD with current major depressive episode; 25, controls	Blood serum (depletion of high abundant proteins)	LC-MS/MS, TMT labeling, validation by MRM (multiple reaction monitoring)	50 proteins altered > 1.5-fold between the two groups	Identified a serum biomarker panel consisting of 6 proteins: Apolipoprotein D, Apolipoprotein B, Vitamin D-binding protein, Ceruloplasmin, Hornerin, and Profilin 1, which could be used to distinguish MDD patients from controls with 68% diagnostic accuracy
^40^	22, first-onset- SZ; 33, controls	Blood serum (depletion of 20 high abundant proteins)	Label-free nano LC-MS/MS (MS^E^), quantification MS intensity	10	Differentially expressed proteins involved in lipid metabolism, cholesterol transport pathway, and immune response
^42^	Sample set 1: 10, SZ; 10, controls Sample set 2: 47, treated SZ; 53, controls	Blood serum (depletion of 20 high abundant proteins)	LC-MS/MS	27 proteins discriminated cases from controls (7 significantly differentially expressed)	Differentially expressed proteins express dysregulation of complement pathway and coagulation cascades
^41^	7, first-onset/drug-naïve SZ; 13, SZ treated with atypical antipsychotic medications; 20, controls	Red blood cells and liver cells	2D-DIGE and shotgun	8	Half of the altered proteins related to oxidative stress. Results suggest that the changes are disease-related rather than a result of drug treatment
^60^	77, SZ (comparing short-term SZ relapse group versus long term-relapse SZ groups)	Blood serum	HumanMAP multiplex immunoassay	Proteins altered associated with symptom severity (6); response prediction (1); time to relapse (8)	Differentially expressed proteins involved in acute phase response, transport, immune response, and glucose metabolic pathways. Suggests biomarkers related to insulin and leptin
^62^	75, pre-proximal SZ; 110, BD; 75 + 110, controls	Blood serum	DiscoveryMAP immunoassay	20	Identified molecules altered prior to clinical manifestations related to inflammation and immune response
^43^	250, first/recent onset SZ; 35, MDD; 32, BD; 329, controls	Blood serum	HumanMAP multiplex immunoassay	34	34 biomarker panel of differentially expressed proteins with diverse functions. SZ cases can be sub grouped based on distinctions in molecular serum profiles
^61^	577, SZ; 110, pre-symptomatic BD; 229, controls	Blood serum	DiscoveryMAP multiplex immunoassay	22	Formed 51-plex biomarker panel with sensitivity and specificity of 83%
^56^	10, euthymic BD-I; 20, depressed BD-I; 15, manic BD-I; 20, controls	Plasma	2DE; MALDI-TOF/TOF MS; Western blotting	32 proteins with 1.5-fold changes	BD pathophysiology may be associated with early perturbations in lipid metabolism that are independent of mood state, while CA-1 may be involved in the pathophysiology of depressive episodes
^50^	Cohort 1: 23, first-onset MDD; Cohort 2: 15, first-onset MDD; 42 + 21, controls	Blood serum	HumanMAP multiplex immunoassay	11	Evidence for an increased pro-inflammatory and oxidative stress response, followed by a hyperactivation of the HPA-axis in the acute stages of first-onset MDD, as well as dysregulation in growth factor pathways
^44^	112, SZ/SZA/schizophreniform; 133, asymptomatic siblings; 87, controls	Blood serum	Human DiscoveryMAP multiplex immunoassay	10	Findings suggest presence of a molecular endophenotype involving disruption of insulin and growth factor signaling pathways as an increased risk factor for SZ. Of 10 differentially expressed proteins, 5 showed significant differences in symptomatic siblings and 5 were altered in asymptomatic siblings
^45^	42, SZ; 46, controls	Plasma	2-D PAGE/MALDI-TOF MS	6	Altered proteins associated with acute phase, coagulation, and transport
^51^	21, first-onset MDD; 21, controls	Plasma (depletion of 7 high abundant proteins)	iTRAQ labeling, 2D LC-MS; 2 peptides/protein, application of decoy database, ELISA for validation	9	Functions of altered proteins primarily involved in lipid metabolism and immunoregulation
^46^	22, SZ; 20, controls	Plasma	2-D PAGE/MALDI	7	Proteins involved in acute phase response and molecular transport further supporting hypothesis of inflammatory response system linked to pathophysiology of SZ

^a^Summary of studies in schizophrenia includes original data abstracted in combination with findings previously reported in reviews by Davalieva et al., (2016) and Nascimento and Martins-de-Souza, (2015)

Of the articles found, 13 were studies on SZ^34–42,44–46,60^, 6 on MDD^18,47–51^, and 5 on BD-I/-II^52–56^ patients, whereas the remaining studies^<57–59,61–62,43^ included a combination of patients of the relevant diagnoses. All studies presented information on up- and down-regulated proteins (see S1). For all studies, information was summarized with regards to first author and year of study, study country, aim, number and sources of participants, sources of information on exposure, medication use, information on comorbidities of included participants, biological sample used, method for protein profiling, confounding factors controlled for, statistical analysis methods, blinding, major findings and outlook. For differentially expressed proteins, outcome measures of interest included: statistical significance, fold change and direction of differential abundance, and associated pathways and processes.

For each differentially expressed protein, we used the UniProt Knowledgebase to obtain entry codes and recommended names and ascertained the overlap across the three diagnoses^63^. Throughout this review we refer to proteins by their recommended abbreviations. For full protein names and explanations, please refer to supplementary table 2 (see S2). As examples of the utility of findings from these studies, we have evaluated the abstracted data in three different ways by (1) summarizing the overlap (specificity) of differentially expressed proteins, (2) cross-referencing proteomic findings with genetic loci identified in genome-wide association studies (GWAS), and (3) identifying the top enriched canonical pathways for each diagnosis via Ingenuity Pathway Analysis (IPA; QIAGEN, Inc., https://www.qiagenbioinformatics.com/products/ingenuitypathway-analysis)^64^. Bioinformatic enrichment analyses were also carried out with Gene Ontology annotations (*P* < 0.05 after Bonferroni correction for multiple testing) to confirm IPA results using PANTHER GO-Slim Biological Process, PANTHER Protein Class and PANTHER Pathways analysis^65^. The Homo sapiens reference list containing all genes as provided by default in PANTHER was used as the comparison background group for enrichment analyses.

## Heterogeneous study designs

Our appraisal of proteomics studies reflects the heterogeneity in approaches used for biomarker identification of psychiatric disorders. Sample sizes ranged from 7^41^ to 687^18^ patients. All but two studies included healthy controls (HC) for comparison. One study^48^ compared patients with a diagnosis of remitted later life depression (LLD) with mild cognitive impairment to patients with normal cognitive function. A second study^60^ contrasted short-term SZ relapse patients with long-term SZ relapse patients at two different time points. Methods for protein depletion and quantification varied. For example, some studies used depletion of high abundant proteins by antibody-based methods prior to the proteomic analysis. The way these depletion methods are currently used, often results in irreproducibility of protein quantification, especially for low abundant proteins^66^. Most groups used label-free quantification, but some applied chemical labeling techniques like iTRAQ or TMT, which allow multiplexing and therefore a higher sample throughput, albeit at the cost of ratio distortion.

Sample storage differences and time-wise storage limitations are critical when considering the results presented by proteomic studies. Measuring protein analytes is a delicate task as samples are alive and can change due to molecular responses to changing conditions. Therefore, it is critical that consistency in sample storage and treatment, as well as sample documentation is kept. Even with optimal preparations, “proteomic change can occur resulting in individual outlier samples or overall drift,” therefore quality markers in combination with careful sample documentation is crucial^67^. Unfortunately, these critical details are not often reported but must be kept in mind when comparing results across studies.

Differences in sample sizes, tissue type sample preparation, analytical pipeline, mass spectrometry instruments, data analysis, and statistical approaches used, as well as heterogeneity in case definitions, made evaluation between studies difficult. These inconsistencies in workflows is an issue inherent to formal comparisons of proteomics data, especially for performing meta analyses^68^. While some studies used matched case-control approaches and performed more basic t-tests for group comparisons, others used regression modeling which enabled inclusion of confounding covariates. Although several studies accounted for multiple testing, others failed to do so which would undoubtedly led to a number of false positive proteins having been identified as differentially abundant. While some studies used stricter significance thresholds (*p* < 0.01) others used (*p* < 0.05) without correcting for multiple testing. Furthermore, some studies have chosen to only report those proteins that surpassed a specific fold-change threshold and therefore data on those proteins with smaller effect sizes may not be well represented and those with lower fold change thresholds may be contributing to false positive results. Differences in patient populations and selection criteria also confounded inter-study comparisons. Moreover, multiple studies^39,41,42,45–47^ failed to disclose whether critical confounders, such as diet and smoking, were considered. As blood is a highly dynamic tissue, in contact with nearly every tissue of the body, it reflects a number of external factors that need to be controlled for^13^. Recent work has shown that robust workflows can be developed, which accurately take these factors into account^30^.

## Summary of study results

**Fig. 2:**
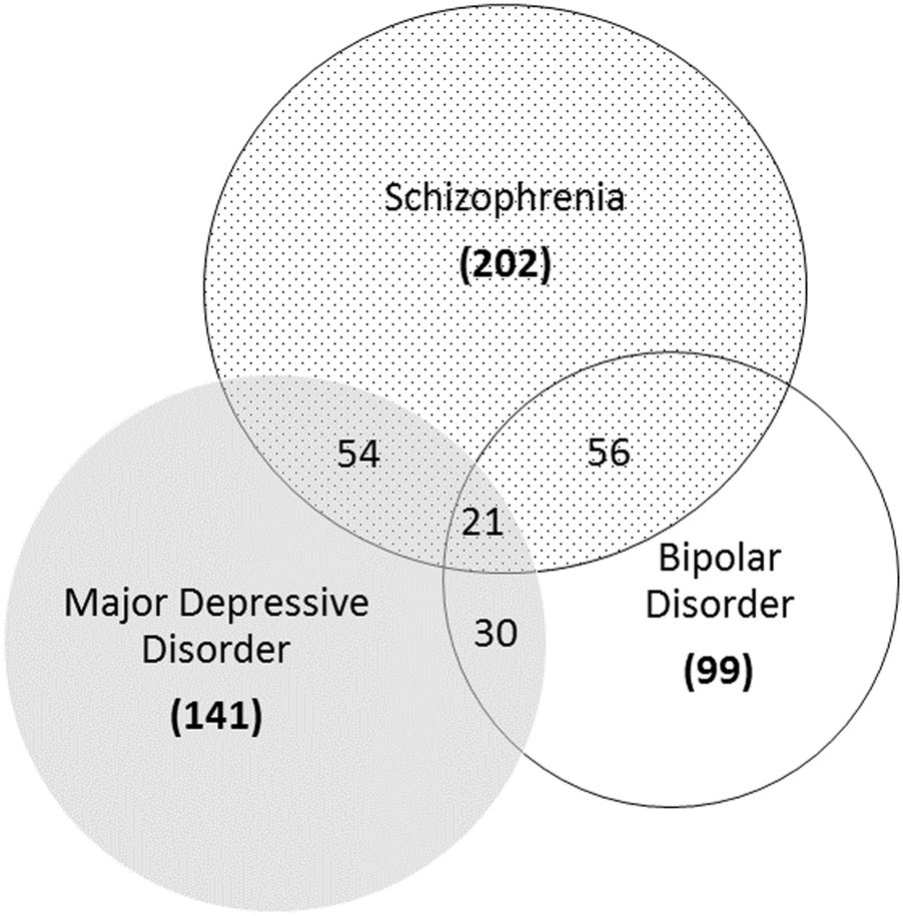
Venn diagram depicting the number of differentially expressed proteins and small molecules found in schizophrenia (SZ), major depressive disorder (MDD), and bipolar disorder (BD) patients for all studies

In total, 323 protein (respectively peptide) signals were differentially abundant; 202 linked to SZ, 141 to MDD, and 99 to BD (see Fig. 2). For SZ, increased circulating levels of insulin-related peptides were frequently reported. Several interleukins, namely IL10, IL12B, IL17A, and IL5, and growth factors such as BDNF, were differentially expressed in SZ patients as reported by at least two independent studies. Several studies reported dysregulation (often a reduction) of apolipoproteins, with at least 2 studies having reported dysregulation of APOA1, APOA2, APOA4, and APOC1. Two studies investigated the potential of assay panels for the differentiation of SZ patients from controls. Schwarz et al. identified a set of analytes, reproducibly altered in SZ patients compared to healthy controls. The refined 51-plex immunoassay had an overall sensitivity of 83% and specificity of 83% with a receiver operative characteristic area under the curve (ROC-AUC) of 89 %^61^. Another study by Schwarz, Guest and Rahmoune et al. identified a signature of 34 analytes and performed a partial least squares discriminant analysis which gave a separation of 60–75% of SZ patients from controls across five independent cohorts. The same analysis gave a separation of ~50% of MDD patients and 10–20% of BD subjects from controls^43^.

For BD, one study reported intriguing findings of a combination of 20 significantly altered proteins/metabolites, including cortisol, CTGF, APCS, and TFF3 prior to clinical manifestations^62^. From this study, Schwarz et al. concluded that their findings could be potential biomarkers for incorporation into diagnostic tests to help identify vulnerable patients early in the disease process^62^. Another study by Alsaif et al. highlighted the differences in proteome coverage/reliability of measurement in a cohort of BD patients and HC. They showed that distinct molecules were measured with marked differences in variation in serum and plasma and acknowledged that variations in measurements could potentially obscure actual differences in data sets^52^. A study by Frye et al. assessed the feasibility of MAP in distinguishing bipolar patients from HC and differentiating subgroups of mood disorders. They found that GDF15, RBP4, and TTR were good predictors of BD-I with an ROC AUC of 0.81. Protein levels of GDF15, HPX, NPN, MMP7, RBP-4, and TTR were higher in BD-I versus unipolar and BD-II patients, as well as controls^53^. One study of BD reported differential abundance of molecules involved in cell death/survival pathways^55^ while another concluded BD pathophysiology may be associated with perturbations in lipid metabolism^56^. Notably, APOA1 and APOL1 were differentially expressed independent of mood state^56^.

Multiple studies of MDD identified differential abundance of pro-inflammatory and oxidative stress response proteins. Stelzhammer et al. reported a change/correlation of ACE, acute phase proteins, BDNF, C4B, cortisol, cytokines, growth hormone and SOD1 with symptom severity^50^. Chen et al. reported three differentially expressed complement proteins validated with ELISA; C3, MDD > BD-II > HC; CFI and C4BPA, HC > MDD > BD-II subjects^57^. Diniz et al. investigated proteins associated with cognitive impairment in later life depression and reported higher levels of CCL13, CXCL11, CCL18, and lower levels of IL12B; reduced levels of KITLG; reduced levels of IGFBP3 and IGFBP5^48^. Xu et al. also found altered proteins involved in immunoregulation and lipid metabolism^51^. Bot et al. found analytes related to cell communication and signal transduction (PPY, MIF, S100A12, IL1RN, and TNC), immune response (CXCL1) and coagulation (VWF)^18^. These alterations were associated with acute depression symptomatology. One study found insulin to be the marker with the highest statistically significant finding, increased in MDD cases compared to controls^58^. Lee et al. identified a serum biomarker panel of six proteins (APOD, APOB, GC, CP, HRNR, and PFN1) which could distinguish MDD patients from HC with a 68 % diagnostic accuracy^49^. Another study attempted to identify any protein peaks that allowed for the distinction of patients from controls and by taking the three signals with greatest potential as candidate biomarkers were able to identify patients with limited false positive identifications (AUC = 0.92)^47^.

There was a slight overlap of differentially expressed proteins across studies (See S2). Twenty-one altered proteins and small molecules overlapped across all three diagnoses in at least one of the studies: A2M, APOA1, APOA2, APOB, APOC1, APOH, C4BPA, C3, CSF2, IgM, KNG1, KITLG, LH, MIF, progesterone, TF, APCS, TTR, CD40, GC, and PROS1. Table 2 reports those proteins that were differentially abundant in the same direction as reported by at least two studies for each disorder. Three of these proteins involved in immune response, Compliment C3 (up), Macrophage Migration Inhibitory Factor (up), and Immunoglobulin M (down) were differentially abundant in the same direction across all three disorders. Many more proteins showed overlap between two disorders or were changed in abundance similarly specifically for one disorder. These findings are in agreement with the widely accepted assumption that no single biomarker for diagnoses of severe mental illnesses exists but rather a panel of biomarkers will be necessary for clinical application.

**Table 2:** Proteins differentially abundant in the same direction across disorders

Protein	Schizophrenia	Bipolar disorder	Major depressive disorder	Up/Down
Adiponectin	X			Down
Alpha-1-antitrypsin	X		X	Up
Alpha-2-HS-glycoprotein	X			Down
Alpha-2-macroglobulin		X	X	Down
Angiogenin			X	Up
Apolipoprotein A-I		X	X	Down
Apolipoprotein A-II	X			Down
Apolipoprotein C-I	X			Down
Apolipoprotein D			X	Up
C4b-binding protein alpha chain		X		Down
Carcinoembryonic antigen-related cell adhesion molecule 5	X			Up
C-C motif chemokine 5	X			Up
Chromogranin-A	X			Up
Complement C3	X	X	X	Up
Complement factor B	X			Up
Connective tissue growth factor	X			Up
Cortisol	X			Up
Epidermal growth factor receptor			X	Up
Glutathione-S-transferase A3	X	X		Up
Glycoprotein hormones alpha chain (CGA)			X	Down
Granulocyte-macrophage colony-stimulating factor	X			Down
Hemopexin		X		Up
Immunoglobulin M	X	X	X	Down
Insulin-like growth factor-binding protein 5			X	Down
Interleukin-1 receptor antagonist protein			X	Up
Interleukin-12 subunit beta (IL12B)	X			Down
Kit ligand		X		Down
Macrophage migration inhibitory factor	X	X	X	Up
Matrilysin (MMP7)		X		Up
Matrix metalloproteinase 9	X		X	Up
Plasminogen activator inhibitor 1	X			Up
Protein S100-A12			X	Up
Resistin	X			Down
Serum amyloid P-component	X			Up
Sex hormone binding globulin		X		Down
Somatotropin (GH1)			X	Down
Sortilin	X			Down
Thyroxine-binding globulin	X			Up
Vitamin D-binding protein			X	Up

## Similarities with GWAS findings

In common with GWAS findings, the genes coding for complement factors C3 and C4-related molecules, as well as Inter-Alpha-Trypsin Inhibitors Heavy Chain (ITIH) 1, 3 and 4 (whose levels in peripheral blood are altered in SZ, see Table S2), were associated with an increased risk for this disorder^69,70^. The observation of changes in the expression levels of *C3* and *C4*-related molecules in peripheral blood in proteomic studies is especially interesting in the case of SZ. The most compelling genetic association reported by genome-wide association studies in this disorder has been identified in the *C4* locus in the *major histocompatibility complex* (*MHC*; chromosome 6)^69,70^. Genetic variation at this locus influences the mRNA levels of C4A in the brain, highlighting the interest of *C4* and *C3*, which are functionally related, as potential peripheral biomarkers. However, as key molecules in innate immunity, C3 and C4 levels are altered in many infectious and low level inflammatory conditions as well. Furthermore Inter-Alpha-Trypsin Inhibitors Heavy Chain 1, 3 and 4, whose levels change in SZ (Supplementary Table 2) are encoded by genes (*ITIH1*, *ITIH3*, *ITIH4*) mapping to a gene cluster locus in chromosome 3, which also shows genome-wide association with regards to SZ risk^70^. With regards to genome-wide associated *loci* in BD or MDD, we found no further overlaps with the differentially expressed proteins. Ongoing GWAS studies in these disorders based on ever-increasing samples may uncover further overlaps between genetic risk loci and the peripheral proteome in these patients.

## Associated processes and pathways

The top five enriched canonical pathways for each diagnosis, based on the aforementioned proteome data, as determined by IPA are listed in Table 3. The top hits were FXR/RXR Activation (*p* = 2.89E-30), Acute Phase Response Signaling (*p* = 2.90E-31), and LXR/RXR Activation (*p* = 1.62E-23) for BD, SZ, and MDD, respectively. Diagnostic–specific enrichment analyses using GO determined significant overlap in enriched biological processes across diagnoses supporting IPA results of enrichment of pathways involved in immune and inflammatory responses (See S3-S11). The top biological processes across all three disorders included response to interferon-gamma, the cytokine-mediated signaling pathway, locomotion, blood coagulation and complement activation. Significant PANTHER pathways for all three diagnoses were the blood coagulation, plasminogen activating cascade, and interleukin signaling pathways. Enrichment of protein classes included the complement component, chemokine, and growth factors classes for all three diagnoses.

**Table 3:** Diagnosis specific top canonical pathways according to ingenuity pathway analysis (IPA)

	*p*-value	Overlap
**Diagnosis: bipolar disorder**		
FXR/RXR activation	2.89E-30	17.5% 22/126
LXR/RXR activation	7.69E-29	17.4% 21/121
Acute phase response signaling	3.12E-27	12.9% 22/170
Clathrin-mediated endocytosis signaling	8.20E-14	7.0% 14/199
Atherosclerosis signaling	1.60E-13	9.4% 12/127
**Diagnosis: schizophrenia**		
Acute phase response signaling	2.90E-31	17.1% 29/170
LXR/RXR activation	7.01E-31	25.1% 26/121
FXR/RXR activation	1.22E-25	18.3% 23/126
Hepatic fibrosis/hepatic stellate cell activation	1.63E-24	13.7% 25/183
Atherosclerosis signaling	1.56E-22	16.5% 21/127
**Diagnosis: major depressive disorder**		
LXR/RXR activation	1.62E-23	16.5% 20/121
Acute phase response signaling	6.89E-22	12.4% 21/170
FXR/RXR activation	1.38E-21	15.1% 19/126
Agranulocyte adhesion diapedesis	9.50E-17	9.4% 18/191
Granulocyte adhesion diapedesis	6.27E-16	9.5% 17/179

*Note*: Proteins identified as differentially expressed across diagnoses were uploaded to IPA in order to identify enriched pathways according to present knowledge. Given *p*-values correspond to the likelihood that the association between a set of proteins and a given pathway is due to random chance. The *p*-value was calculated using the right-tailed Fisher Exact Test

The enriched pathways observed have previously been implicated in disease pathology: immune/inflammatory response, metabolic and hormonal pathways in SZ;^11,25,71^ the immunologic hypothesis in MDD;^13,14^ and energy metabolism, as well as oxidative stress and inflammatory response in BD^72^. Clearly these enriched pathways are quite similar across diagnoses and are also involved in other conditions. This raises questions about the specificity and validity of the results of the studies surveyed here. These findings demonstrate that current data are unable to truly distinguish between different diseases. While numerous potential biomarkers have been identified, their pathophysiological significance remains unknown and their practical clinical application limited^19^. We believe that this is due to the intrinsic challenge of discovering changes in peripheral protein abundance that accurately reflect molecular alterations in the brain, as well as the very high technological requirements of in depth and accurate analysis of the plasma proteome. Additional biomarker research using the latest state-of-the-art proteomics technology is promising for the future.

## Perspectives

It is well recognized that the current nosological framework represented by the DSM-IV and ICD-10 has serious shortcomings with respect to validity and that there is a need for a classification system incorporating measures at various levels of analysis ranging from genes and cellular/molecular mechanisms to behavioral and clinical measures^73^. To move away from “signs and symptoms” -based diagnoses, biological or physiological markers need to be identified that can be used to re-organize the current systems of classification for improved diagnosis and stratification of patients. This has the potential to inform the type, time, and course of interventions and would allow disorders to be subtyped based on physiological criteria, thus leading to a more biologically grounded and precise approach to psychiatric treatments^20^. Biomarkers that can reliably be detected in the bloodstream would enable minimally invasive and economical monitoring of patients at progressive disease stages and treatment courses^71^. Therefore, high-throughput proteomics approaches in principle offer a powerful tool for research in severe mental illness.

Proteomic studies of other neuropsychiatric disorders have exposed the promise of proteomic technologies for biomarker identification and disease tracking. For example, Lee et al. were able to identify 18 proteins expressed in blood that were approximately 90 % accurate in differentiating Alzheimer’s patients from HC. They were able to predict disease course in terms of cognitive impairment with this information^74,75^. Protein biomarkers have also been identified for the pathological status of patients with Huntington’s disease where clusterin (apolipoprotein J) was reported as a promising candidate^76^.

**Fig. 3:**
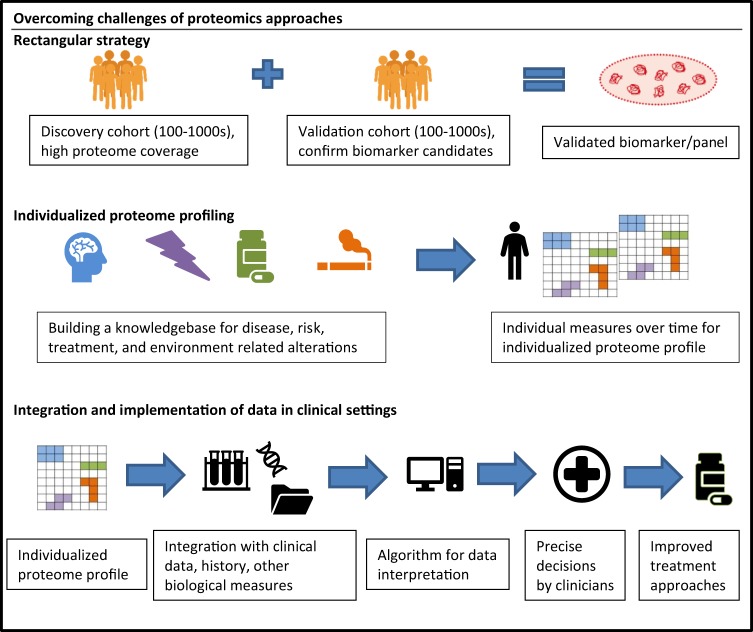
Schematic for overcoming challenges of proteomics for biomarker discovery in psychiatry

Currently, the costs of proteomics studies are quite high, comparable to those of gene expression or genomic analyses several years ago. Similarly, to those technologies, proteomic workflows will need to be made more cost effective, a development which is already underway. Likewise, the limited reproducibility of many proteomic workflows can be overcome by modern proteomic pipelines^26^. In the past, the lack of reproducibility of many of the biomarker findings in independent studies has resulted in ambiguous and conflicting results due to the above mentioned methodological issues or patient heterogeneity^12^. Modern proteomics workflows enable high-throughput studies with large cohorts of well-defined samples (see Fig. 3)^23^.

Accurate selection of the clinical population, sampling time, and standardization of procedures for sample processing are critical for any successful biomarker studies, including proteomics. Sample size also needs to be considered to enable sufficient statistical power to be maintained after stratification for potential confounding factors (i.e., diet, smoking, alcohol use, exercise)^13^. Future studies, and in the best case longitudinal in design, would be ideal as they would allow for repeated measures for validated results and conclusions and because they automatically account for inter-individual differences in protein expression levels. In addition, results from discovery proteomics should be validated in larger cohorts. As is the case in GWAS, proteomic studies with sizable discovery, as well as validation cohorts would have great advantages in actually finding and confirming biomarkers (‘rectangular biomarker strategy’)^23^.

To support progress in the field of psychiatric proteomics, further advances in proteomic profiling techniques are needed. Evidence of the lack of very reproducible, robust, and high-throughput proteomic workflows to identify and verify potential biomarkers in large cohorts have been hampering the field over the last years. However, recent results show that it is possible to introduce more rapid and robust proteome profiling pipelines^30,77^. Additionally, advances in targeted proteomic pipelines for absolute protein quantification are needed to study well defined marker proteins in a highly reliable and reproducible fashion using heavy isotope labeled standards^78^.

With rapid growth in the omics fields, vast quantities of data are being produced. Therefore, it is important to consider efficient data-mining technologies, as well as the establishment of international public accessible databases like PRIDE (http://www.ebi.ac.uk/pride/archive/)^12^. Applied to clinical proteomic studies, this would allow for multicenter collaborations to combine large-scale data from multiple levels of analysis, using standardized nomenclature and integration with other databases^12^. In our review, even protein reporting often lacked approved nomenclature for gene symbols, making between-study comparisons difficult, which would be avoided by using UniProt Knowledgebase^79^ entry codes and recommended names^80^. Efforts to integrate peripheral blood profiling data with other laboratory and clinical endpoints have the potential for the identification of novel ‘multidimensional’ markers and to reveal novel insight in the classification of complex diseases^13^. It is evident that such a joint effort requires interdisciplinary collaborations including biochemists, biologists, molecular genetics, as well as statisticians and bioinformaticians alongside clinicians to draw valid, reliable conclusions.

## Conclusion

Here we have uncovered some of the pitfalls and potential of proteomics studies for understanding complex psychiatric disorders. With support from genomics, interesting pathways have already been implicated, especially for SZ, thanks to big sample sizes. Further efforts towards establishing prospective cohorts within a big data framework will contribute greatly towards the success of future proteomic studies of psychiatric disorders. While various drawbacks have hindered investigations in the last decades, recent improvements for more rapid and robust methods have introduced a huge potential that is yet to be exploited. Blood, plasma and serum are still untapped source of possible biomarkers which have potential to impact not only clinical care but also the conduct of drug trials in the future^13^. They will greatly complement other unbiased–omic approaches in the quest for progress of psychiatric research of severe mental disorders.

## Electronic supplementary material

S1-Compiled results

S2-Results by protein

S3-SZ biological process

S4-BD biological process

S5-MDD biological process

S6-SZ pathways

S7-BD pathways

S8-MDD pathways

S9-SZ protein class

S10-BD protein class

S11-MDD protein class

Supplemental legends
