# Supplementary material for: Proteomics for blood biomarker exploration of severe mental illness: pitfalls of the past and potential for the future
Source: Transl Psychiatry. 2018 Aug 16;8:160. doi: 10.1038/s41398-018-0219-2 (PMC6095863; doi:10.1038/s41398-018-0219-2)
Supplement: Supplementary file 12 — Supplemental legends [file 41398_2018_219_MOESM12_ESM.doc]

S1. Differentially expressed proteins reported by included studies

S2. Comparison of differentially expressed proteins across diagnoses

S3. Enriched biological processes in schizophrenia

S4. Enriched biological processes in bipolar disorder

S5. Enriched biological processes in major depressive disorder

S6. Enriched pathways in schizophrenia

S7. Enriched pathways in bipolar disorder

S8. Enriched pathways in major depressive disorder

S9. Enriched protein classes in schizophrenia

S10. Enriched protein classes in bipolar disorder

S11. Enriched protein classes in major depressive disorder
